# On the Internal Use of Turpentine Oil in Cases of Hemorrhage

**Published:** 1848

**Authors:** L. Percy


### ARTICLE XIV.

*On the Internal Use of Turpentine Oil in Cases of Hemorrhage.*
By L. Percy, M. D.

The author, after noticing the fact that several writers—Adair, Nicol, Johnson, Warneck, Copland, Ashwell, and Pereira—have spoken of the efficacy of the essential oil of turpentine in hemorrhagic diseases, observes that this remedy seems nevertheless to be little used by practitioners. In the cases in which he first made trial of it, hæmaturia of two years' standing, in an old man of eighty, was stopped in 24 hours by eight drops of oil of turpentine, and did not return. He has since used it in cases of hemorrhage, and always with a favorable result. The cases in which its use is indicated are those of passive hemorrhage. It must not not be employed in cases where there is an active determination of blood, and where the pulse is full. When the discharge of blood is the cause of organic disease, as of disease of the uterus, or of tubercular disease of the lungs, the action of the remedy is not so efficacious; but the author has seen a case of scirrhus of the womb, in which the hemorrhage was for some time stopped by this remedy. The author has found the action of turpentine oil very rapid, an effect being manifest in a few hours, often after one small dose. In order to better ascertain its power, he used it alone, without having recourse to local astringents or cold applications, where he could do so without fear of endangering the life of the patient. He has used it most frequently in cases of menorrhagia and epistaxis; but he mentions, that it appears to him to be particularly applicable in cases of hemorrhage attending typhus. He noticed the fact that turpentine exerts different actions on the body according as it is taken in large or small doses, being more readily absorbed in the latter case; as he remarks, that as its beneficial action in cases of hemorrhage must depend on its being absorbed, the inference would be drawn, that the doses in which it is given in such cases ought to be small. His experience confirms this conclusion. He has always found a dose of from eight to thirty drops sufficient. The best vehicle for it is almond emulsion, with a little gum arabic. When there is pain in the abdomen, a few drops of laudanum may be added.—*London Med. Gazette in Ibid.*

